# Multi-Robot 2.5D Localization and Mapping Using a Monte Carlo Algorithm on a Multi-Level Surface

**DOI:** 10.3390/s21134588

**Published:** 2021-07-04

**Authors:** Vinicio Alejandro Rosas-Cervantes, Quoc-Dong Hoang, Soon-Geul Lee, Jae-Hwan Choi

**Affiliations:** 1 Mechanical Engineering Department, Kyung Hee University, Yongin 17104, Korea; viniciorosas@hotmail.es (V.A.R.-C.); hoangquocdong.vimaru@gmail.com (Q.-D.H.); choisida89@gmail.com (J.-H.C.); 2 Integrated Education Institute for Frontier Science and Technology (BK21 Four), Kyung Hee University, Yongin 17104, Korea

**Keywords:** multi-robot, localization, 2.5D mapping, Monte Carlo algorithm, multi-level surface

## Abstract

Most indoor environments have wheelchair adaptations or ramps, providing an opportunity for mobile robots to navigate sloped areas avoiding steps. These indoor environments with integrated sloped areas are divided into different levels. The multi-level areas represent a challenge for mobile robot navigation due to the sudden change in reference sensors as visual, inertial, or laser scan instruments. Using multiple cooperative robots is advantageous for mapping and localization since they permit rapid exploration of the environment and provide higher redundancy than using a single robot. This study proposes a multi-robot localization using two robots (leader and follower) to perform a fast and robust environment exploration on multi-level areas. The leader robot is equipped with a 3D LIDAR for 2.5D mapping and a Kinect camera for RGB image acquisition. Using 3D LIDAR, the leader robot obtains information for particle localization, with particles sampled from the walls and obstacle tangents. We employ a convolutional neural network on the RGB images for multi-level area detection. Once the leader robot detects a multi-level area, it generates a path and sends a notification to the follower robot to go into the detected location. The follower robot utilizes a 2D LIDAR to explore the boundaries of the even areas and generate a 2D map using an extension of the iterative closest point. The 2D map is utilized as a re-localization resource in case of failure of the leader robot.

## 1. Introduction

For mapping and localization on an uneven or multi-level surface, 3D LIDAR is needed. However, the price of a 3D LIDAR is very high, and the required computing power and resources are also high. A single small robot cannot process both 3D localization and mapping for real-time navigation. Since our previous work [1] studied mapping and localization on uneven surfaces, we decided to extend the experiment using a second robot. The mapping and exploration time is simplified, and the computational time is improved.

The present experiment intends to provide a starting point for a bigger multi-robot team, where the more robots, the faster the mapping task could be performed.

Multi-robot mapping and exploration encounter sparse features in large environments. Alternatives for 2.5D and 3D mappings include 2D–3D feature matching [2] and online point cloud segmentation. Multi-robot systems reduce the mapping time, improve map accuracy and robot localization [3,4,5]. The iterative closest point (ICP) and the random sample consensus (RANSAC) methods are solutions for point cloud registration, but those approaches have issues with the local minimum. Merging image-key points into the point cloud can also reduce the local minimum error [6]. LIDAR odometry and mapping (LOAM) [7] uses point features and edges-planes for scan registration but lacks loop closure in large-scale maps.

Further, scale mismatch and repeating patterns are common issues in robot mapping. A solution for those challenges involves fixing the coordinate transformations and minimizing the distance in input maps. Optimizing point registration [8,9] and using image registration [10] reduce scan misalignments. However, those methods lack an initial guess and local minima. For multi-robot simultaneous localization and mapping (SLAM), idleness optimizes patrol activities [11], and digital pheromones could provide event relevance [12]. The Hough transform uses rotation and translation to divide the mapping space [13]. Bayesian mapping [14] and open-source data [15] enhance SLAM. Mielle et al. [16] employed SLAM in extreme conditions. In an open space, topological representation and maximal common sub-graphs (MCS) allow for fast robot localization [17]. The vertices contained in metric space updates the robot paths. The Voronoi diagram [18,19], segmented regions matching [20], and environment metrics [21] aid map alignment.

The occupancy grid map plays a vital role in the context of multi-robot map representation. The said map upgrades each cell using the inverse sensor model. Multi-robot mapping and exploration have already been addressed in several research approaches [22,23]. Subsequently, the inverse sensor model evaluates the laser scan as a vector. Graph matching aligns the occupancy grid with floor maps [20]. Kaufman et al. [24] measure 2D distances using laser scan ray casting. The occupancy grid and a Monte Carlo algorithm calculate the 2D pose uncertainty [25]. Gaussian modeling has applications for the occupancy grid [26], mapping, and point cloud modeling [27]. A learning trial [28] and a Bayesian occupancy map also employ an inverse sensor model [29,30]. Despite the robustness of grid-based map representation, this method depends on the inverse sensor model and is sensitive to simulation errors.

In a volumetric representation of 3D space, map simplification is critical for robot mapping. Approaches such as the adaptive online method and voxel compression divide the map [31,32]. Octomap reduces the mapping framework [33,34], and a robot-centric grid enhances the map resolution [35]. Furthermore, communication enhancement is critical for multi-robot systems. Cieslewski et al. [36] propose a decentralized visual SLAM by encoding the environment information to reduce communication requirements. A coordinated multi-robot exploration under connectivity constraints is presented in [37], where each robot keeps connectivity with the teammates. Smith et al. [38] use distributed inference-based communication for a 3D space. The main problem in centralized and decentralized multi-robot exploration is map merging and robot localization. [4,13]. To establish proper communication, we utilize a decentralized system after considering the approaches in the recent years.

Deep learning and machine learning have become trends for robot mapping and localization in the last few years. Through trial and error, robots can adjust their behavior in a dynamic environment. In a multi-robot real-time obstacle avoidance [39], the authors propose a continuous domain detection for obstacle avoidance. An actor-critic component with specific training allows for obstacle avoidance on multi-robot systems [40]. Those techniques entail cooperative interactions. For object detection, methods as You only look once (YOLO) and SSD [41,42] use a single convolutional neural network (CNN) to detect the target position and properties. Approaches such as those in [43,44] use faster reinforce convolutional neural network (Faster R-CNN) for object detection. Our work also employs Faster R-CNN for object detection because of the speed detection accuracy under our experimental conditions.

Besides the limitations of mapping, localization, scan alignment, and map representation, multiple robot systems must adapt to new architectural environments. Nowadays, slopes commonly occur to facilitate transit from different areas in houses, warehouses, and buildings. Thus, having a robust localization in multi-level space is critical. Multi-level areas represent a challenging scenario wherein a mobile robot may lose balance, and 2D LIDAR information provides a vague reference for localization.

Multi-robot systems offer robust and fast field coverage [45]. There is limited work focused on autonomous navigation in sloped and unstructured interior areas, particularly in narrow slopes and crowded spaces. Input from multiple robots renders the system more fault-tolerant than its counterparts. The overlapping of multiple information compensates for sensor uncertainties. Nevertheless, multi-robot systems have limitations in exploring uneven spaces because of the sudden change of flat surfaces [46]. There is extensive work for single and multi-robot systems on 2D flat surfaces. However, robot localization is significantly affected when the robots need to explore areas provided with multiple levels. Thus, the major problem for a multi-robot system involves identifying and finding solutions for multi-robot localization. Multi-robot navigation should be capable of localizing the robot, distinguishing slopes from a staircase, and ascertaining a safe path.

This work proposes a robot framework for cooperative robot navigation in indoor environments with multi-level surfaces. We used two robots to validate the proposed method. The leader robot explores the uneven areas of the multi-level access using a Faster R-CNN to detect indoor ramps. By contrast, the follower robot examines the boundaries of the even areas using 2D LIDAR. We propose a novel 2.5D mapping approach to generate a 2.5D map while the robot is exploring an environment with multi-level terrain. Furthermore, we develop a 2D scan merging method to generate a map and obtain a resource to back up the robot localization. Furthermore, autonomous navigation in uneven and unstructured environments is helpful for mobile robots and provides meaningful information for the design of smart wheelchairs. The remainder of this paper is organized as follows. Section 2 addresses the ramp detection using Faster R-CNN, feature extraction, map merging, and localization. Section 3 provides the experimental results, and Section 4 presents the conclusions.

### System Overview

The proposed experiment uses a base station computer as a manager for the data collection from the two robots. The wheel odometry of both robots constitutes the priority reference for the pose distribution using Monte Carlo localization. We measure the weight of the particles according to the input from each LIDAR. To ascertain the correspondences among the point clouds, we translate them to the given pose from the particle estimation.

**Figure 1:**
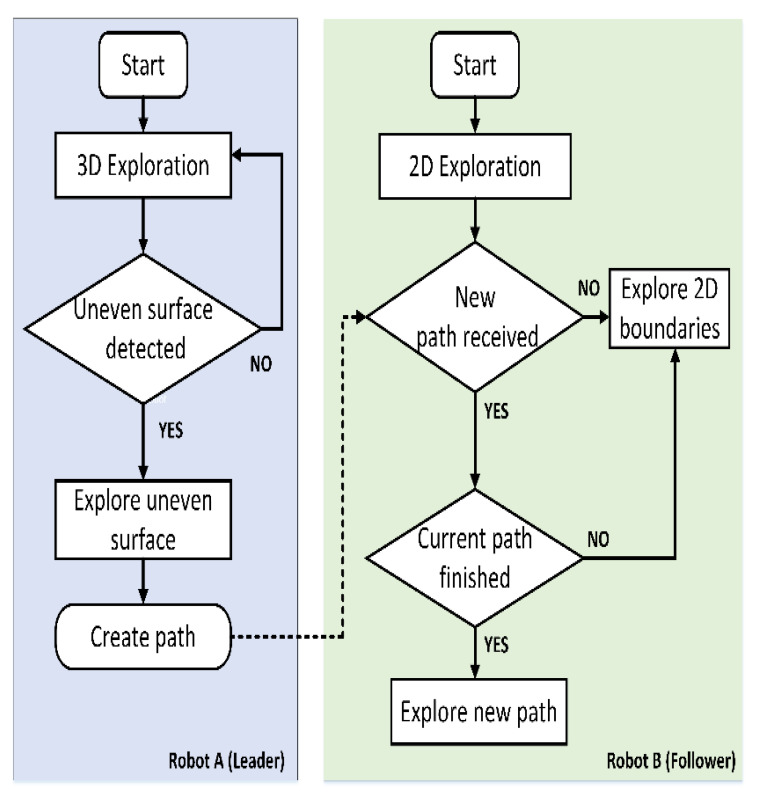
Task flow diagram for the multi robots.

The leader robot generates a global map from the collected LIDAR point clouds. The map can be updated using SLAM according to pose graph optimization and the LIDAR odometry. The follower robot uses the reference generated by the leader robot to identify multi-level access. Once the follower robot enters the new level, it explores all 2D boundaries on the floor. The diagram for the proposed multi-robot system is presented in Figure 1. The follower robot relies on the inertia measurement unit (IMU) to only proceed with the exploration on the X-Y plane. Unless an input of a new path comes from the leader robot, the follower robot will allow the reading in the Y-Z plane.

This work utilizes pose-graph optimization to update a map M. The contributions of this study are as follows: a multi-robot localization according to a Monte Carlo algorithm for multi-level areas, an extension of 2D ICP map merging, and a multi-robot exploration of multi-level areas using a deep CNN.

## 2. Materials and Methods

### 2.1. Faster R-CNN for Indoor Ramp Detection

**Figure 2:**
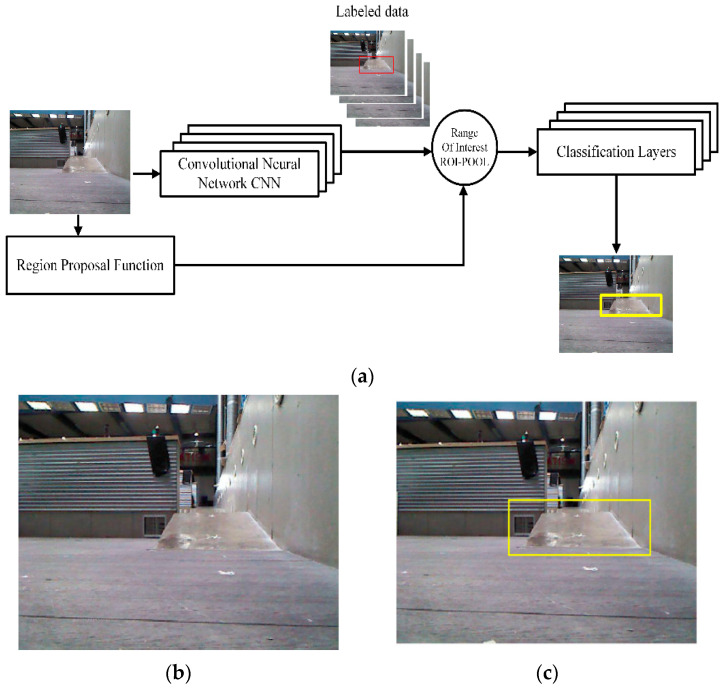
Indoor ramp detection. (**a**) Diagram for Faster R-CNN training. (**b**) Original 2D image obtain by Kinect camera. (**c**) Indoor ramp detected.

Our multi-robot system defines the location of multi-level areas. The leader robot uses a Faster R-CNN [47] for real-time object detection. The Faster R-CNN detector adds a region proposal network to generate region proposals directly in the network, thereby improving object detection. First, training images were collected using a Kinect camera and then resized to a resolution of 224 × 224. Next, each image was divided into grids and assigned a bounding box. A single CNN runs once on every image. The network consists of 15 convolutional layers followed by two fully connected layers. During training and testing, the Faster R-CNN checks the entire image. Figure 2 shows (a) the diagram for the Faster R-CNN training, (b) the 2D image capture by the Kinect camera, and (c) the detected ramp.

### 2.2. 3D Point Clouds and 2D Feature Extraction

The leader robot performs three tasks: detecting uneven areas in the scenery, generating a 3D dense point cloud map, and extracting 2D features from the 3D point cloud.

**Figure 3:**
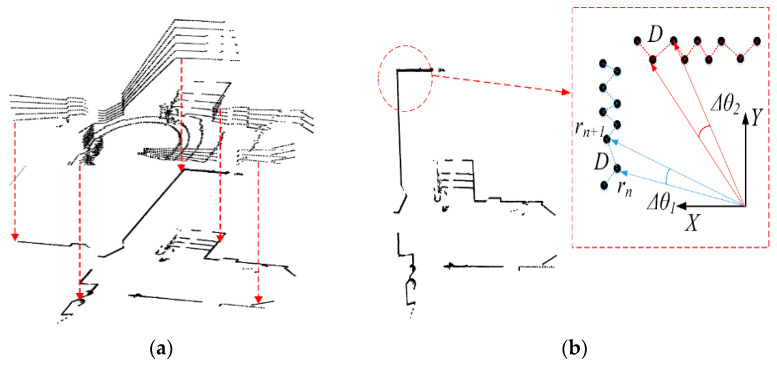
Point splitting method. (**a**) 3D point cloud projection onto the 2D plane. (**b**) Splitting and merging of the 2D edge features.

Based on our previous research [1], the leader robot generates a 2D occupancy grid map $OM_{A}$ using features obtained from a 3D point cloud for localization. The 2D features represent the main edges of the 3D point cloud. Figure 3a shows the projection of the 3D point cloud on a 2D plane. The range of interest (ROI) is set to detect the significant component of the 3D point cloud. To remove the invalid floor points, we employed the Random sampling consensus algorithm (RANSAC). The edge points are denoted as $E_{c}^{1}$ and arranged into polar coordinates. Then, the angle Δθ between the consecutive points $\left(r_{n},r_{n+1}\right)$ of the polar coordinates can be obtained as Figure 3b shows. The distance between those two points is given by Equation (1).

(1) $$D(r_{n},r_{n+1})=\sqrt{r_{n}^{2}+r_{n+1}-2r_{n}r_{n+1}\cos(\Delta \theta )}.$$

Given the 3D LIDAR resolution, the distance threshold is $D_{thd}=0.02\;m$ to split the points. The ROI was divided into segments $Sg_{k\;}\epsilon \;\left\{p_{1},p_{2},\ldots ,p_{N}\right\}$. $\left\{p_{1},p_{2},\ldots ,p_{N}\right\}$ represent the set of points for every segment. The created segments allow us to generate and label features. The orientation for each feature line is Δθ. Figure 3b illustrates how the points were split into segments. Using the described segments $Sg_{k}$ and orientation Δθ we created a set of features $F_{L:A}$.

### 2.3. 2D Mapping and Map Merging

The follower robot explores the flat area boundaries. The set of 2D LIDAR scans collected by the follower robot was divided according to the input received from the leader robot. The leader robot detects a ramp and the follower robot crosses to the ramp, thereby creating a new set of 2D LIDAR scans $s_{L:B}$. Then, using $s_{L:B}$, the follower robot filters each scan using the Voronoi diagram and Delaunay triangulation. Algorithm 1, Line 4 shows the filtering step. After filtering the scans, the follower robot creates an occupancy grid map $OM_{B}$.

We propose an extended version of the ICP for scan merging and further occupancy grid map creation. For every robot pose, $M^{i-1}$ is the reference scan and $S_{c}^{i}$ is the current scan. Given the rotation matrix $R=R_{\theta }$ and the translation t, ICP computes the alignment error *E* between the two datasets and determines the proper rotation R and translation t that minimizes the outcome of Equation (2).

(2) $$E(R,t)=\sum_{k=1}^{N_{r}}\sum_{j=1}^{N_{c}}w_{k,j}{\left\|M_{k}^{i-1}-(RS_{c,j}^{i}+t)\right\|}^{2},$$

where $N_{r}$ and $N_{c}$ are the number of the points in $M^{i-1}$ and $S_{c}^{i}$ respectively. $w_{k,j}$ is 1, if $M_{k}^{i-1}$ is the closest point to $S_{c,j}^{i}$ and is 0 otherwise. ICP rotates the scanned data $S_{c,j}^{i}$ by θ and translates using t to obtain the best alignment to the reference data $M^{i-1}$. We started the mapping and localization matching ${\left[\begin{array}{ccc} 0\; & 0\; & 0 \end{array}\right]}^{T}$ with the coordinate frame. Using ICP scan matching, we obtained a pose correction vector ${\left[\begin{array}{ccc} x_{sm}^{i} & y_{sm}^{i} & \theta_{sm}^{i} \end{array}\right]}^{T}$. The pose correction vector derives the homogeneous coordinate transformation matrix H. By employing the 2D geometric transformation, H was expanded to a 3 × 3 matrix as shown in Equation (3).

(3) $$H=\left[\begin{array}{ccc} \cos\theta_{sm}^{i} & -\sin\theta_{sm}^{i} & x_{sm}^{i}\\ \sin\theta_{sm}^{i} & \cos\theta_{sm}^{i} & y_{sm}^{i}\\ 0 & 0 & 1 \end{array}\right]$$

Then the pose of the robot can be updated by Equation (4).

(4) $$\left[\begin{array}{l} x_{est}^{i}\\ y_{est}^{i}\\ \theta_{est}^{i} \end{array}\right]=\left[\begin{array}{l} x_{est}^{i-1}\\ y_{est}^{i-1}\\ \theta_{est}^{i-1} \end{array}\right]+\left[\begin{array}{l} dx_{i-1}^{i}\\ dy_{i-1}^{i}\\ d\theta_{i-1}^{i} \end{array}\right]+\left[\begin{array}{l} x_{sm}^{i}\\ y_{sm}^{i}\\ \theta_{sm}^{i} \end{array}\right]$$

where ${\left[\begin{array}{ccc} x_{est}^{i-1} & y_{est}^{i-1} & \theta_{est}^{i-1} \end{array}\right]}^{T}$ is the estimated pose of the leader robot t (i−1)−th sampling time, and ${\left[\begin{array}{ccc} dx_{i-1}^{i} & dy_{i-1}^{i} & d\theta_{i-1}^{i} \end{array}\right]}^{T}$ is the difference between the i−th and the (i−1)−th pose of the robot as estimated through the odometer. Scan merging combines the reference data set $M^{i-1}$ and the current data set $S_{c}^{i}$ into a new data set $M^{i}$. Then, we determined the outlier points and validated the workspace scans. Using a sparse point map, we avoid point duplication. The map of the environment was incrementally built according to Equation (5).

(5) Mi=Mi−1∪{(xq,yq)∈Sic|∃(xp,yp) ∈Mi−1: ∥(xq,yq)−(xp,yp)∥ <dth}

where $\left(x_{q},y_{q}\right)$ is a data point of the current scan $S_{c}^{i}$, $\left(x_{p},y_{p}\right)$ is a data point in $M^{i-1}$, and $d_{th}$ is the threshold value for the scan merging. The sensor range determines the proper value $d_{th}$ to achieve optimal scan alignment. The initial state $S_{c}^{i}$ is one “1” and $d_{th}$ is the threshold circle radius.

**Figure 4:**
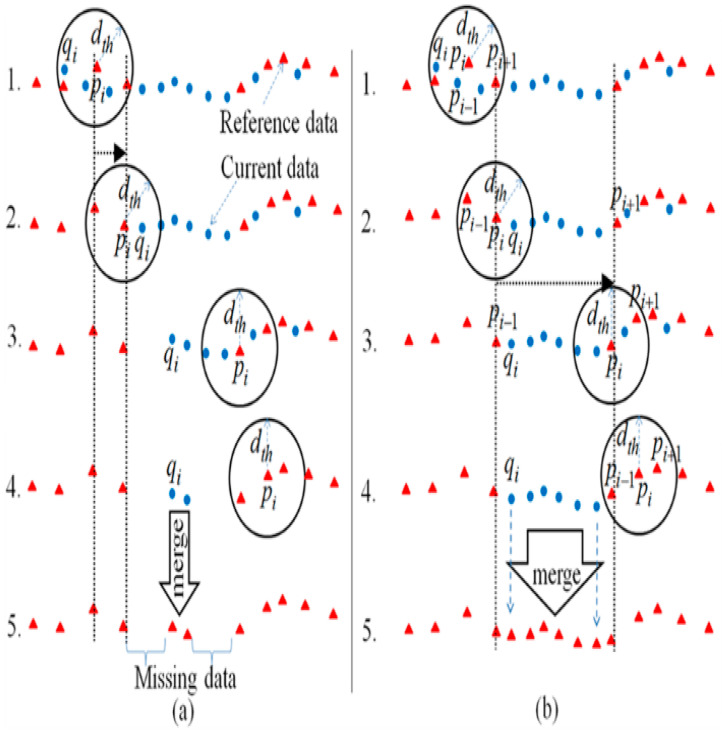
Scan merging. (**a**) Conventional merging rule. (**b**) Modified scan merging rule.

**Figure 5:**
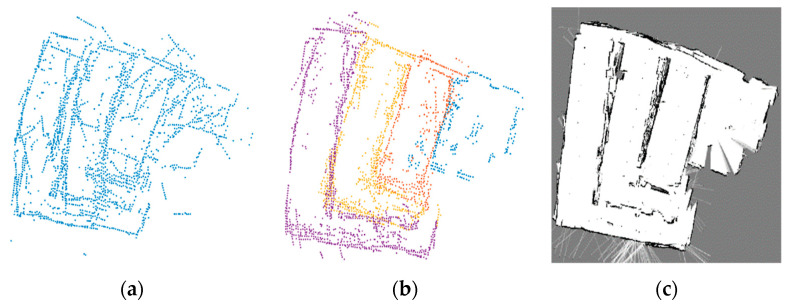
2D LIDAR segmentation and filtering. (**a**) Aligned 2D LIDAR scans without filtering and segmentation (**b**) Segmented and filtered LIDAR scans. (**c**) Generated occupancy grid map.

The merging was executed from left to right, and the threshold circle moves as the data line increases. The new reference $M^{i-1}$ was obtained from the data line “5.” The larger the value $d_{th}$, the smaller the number of points on the map. Figure 4 shows the reference scan $M^{i-1}$ (red triangles) and the current scan $S_{c}^{i}$ (blue circles). Figure 4a presents a circle moving from the previous $P_{i+1}$ line “1” to the new position $p_{i}$ line “2.” Points that do not belong to $p_{i}$ and $p_{i+1}$ are the new scanned points. Figure 4b shows lines “1” and “4” as a duplicated data set and lines “2” and “3” as new scanned points. Our method merges LIDAR scans using a circle threshold that omits adjacent points. If the Euclidian distances between,$p_{i}$, $p_{i-1}$ and $p_{i+1}$ are shorter than the threshold $d_{th}$, then the points are invalid. Algorithm 1, Line 6 includes the described scan merging. Figure 5a–c shows the different stages of the map merging using the segmented data set.
**Algorithm 1 Follower Robot Occupancy Grid Map**$1:\;\mathrm{Input}:\;\mathrm{the}\;\mathrm{follower}\;\mathrm{robot}\;\mathrm{sets}\;\mathrm{local}\;\mathrm{point}-\mathrm{clouds}:\;C_{L:B}$$2:\;\mathrm{Output}:\;\mathrm{Occupancy}\;\mathrm{grid}\;\mathrm{map}:\;OM_{B}$3: for i=1 $\mathrm{to}\;Size\left(C_{L:B}\right)$**do**$4:\;C_{filter\_L:B}\;$$=\mathrm{Filter}(C_{L:B\left(i\right)}$)5: if Leader robot(uneven = 1) **then**$6:\;s_{L:B\left(j\right)}=C_{filter\_L:B\left(i:i+1\right)}$$7:\;S_{L:B\left(j\right)}$$=\mathrm{Merge}(s_{L:B\left(j\right)}$)8: j = j + 19: **end if**$10:\;\;\;OM_{B}\;$*= Occupancy Map*$\left(S_{L:B}\right)$11: **end for**

### 2.4. Communication

**Figure 6:**
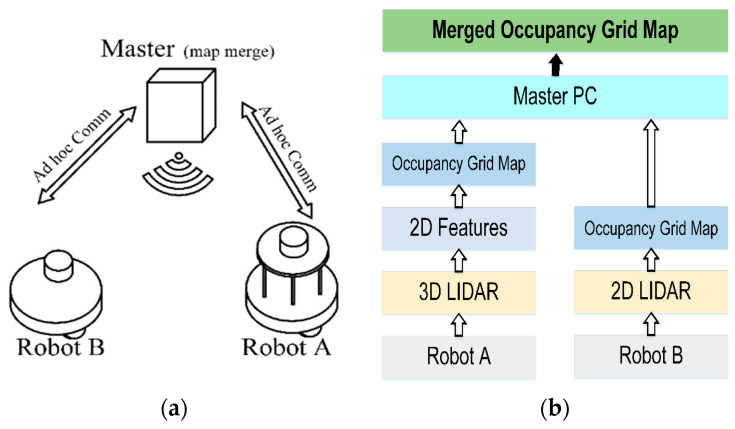
Multi-robot communication. (**a**) Robot A (leader) and Robot B (follower) communication with the master computer. (**b**) Communication flowchart.

To establish multi-robot communication, we used an ad hoc on-demand distance vector (AODV) and a WLAN access point. The AODV is a package of the robot operative system (ROS) and has a unicast and multicast transition. The ROS allows us to create nodes for multi-robot communication. The AODV uses an automatic request datalink to achieve reliable data transfer. The robots do not communicate with each other directly. Instead, the AODV transmits the data using a raw socket, thereby avoiding kernel space. Once each robot has received the data, the master computer publishes the AODV package. With the mentioned protocol and ROS node-publication method, we establish flexible communication among the robots. Figure 6a shows the multi-robot communication diagram. The master station receives a ROS message containing the occupancy grid created by each robot. The master computer then merges the multi-robot trajectories into a single-occupancy grid map. Figure 6b presents the communication flowchart.

### 2.5. Multi-Robot Localization

We propose a multi-robot localization using a Monte Carlo algorithm from our previous study [1]. The leader robot gives the starting point in the initial pose. Each robot uses a sensor reading for particle estimation. We used an iterative process; each robot moves, senses, and re-samples to determine its pose. We can execute a single robot localization when multi-robots have mutual poses. This work assumes that the initial robot pose is known, but each robot does not have global positioning. The leader robot extracts 2D features from the 3D point cloud projection and generates an occupancy grind map $OM_{A}$. The follower robot then uses 2D LIDAR scans to localize itself in the occupancy grid map $OM_{B}$.

**Figure 7:**
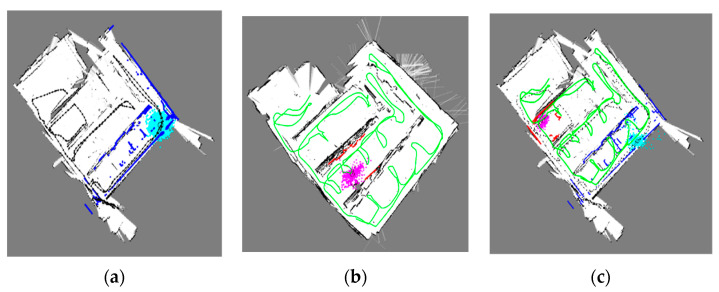
Multi-Robot occupancy maps and trajectories. (**a**) Map of the leader robot, (**b**) Map of the follower robot. (**c**) Map containing leader and follower robots.

The leader robot provides the initial position for localization using the Monte Carlo algorithm. Then, the follower robot proceeds with the localization in the occupancy grid map $OM_{B}$ using the features $F_{L:A}$ described in the Section 2.2 as shown in Algorithm 2, Line 5. To obtain the multi-robot localization, we merge the follower robot trajectory onto the leader robot occupancy-grid-map $OM_{A}$ as shown in Algorithm 2, Line 6. Lastly, we ascertain each robot’s global position in an occupancy grid map. Figure 7 reveals the occupancy map created by each robot: (a) the map for the leader robot, (b) the follower robot map, and (c) the combination of both in one map.
**Algorithm 2 Multi-Robot Localization**$1:\;\mathrm{Input}:\;\mathrm{Set}\;\mathrm{local}\;\mathrm{clouds}\;(C_{L:A}$$)\;\mathrm{in}\;\mathrm{the}\;\mathrm{follower}\;\mathrm{robot}\;\mathrm{occupancy}\;\mathrm{grid}\;\mathrm{map}\;(OM_{B}$)$2:\;\mathrm{Output}:\;\mathrm{Global}\;\mathrm{robot}\;\mathrm{position}:\;R_{G:A}$$,\;R_{G:B}$3:  for i=1 $\mathrm{to}\;N=Size\left(C_{L:A}\right)$**do**$4:\;F_{L:A}$$=Extract\;2D\;Features\left(C_{L:A}\right)$$5:\;OM_{A}$$=Occupancy\;Map\left(F_{L:A}\right)$$6:\;\;\;\;OM_{A}$$=merge(OM_{A},\;OM_{B})$$7:\;\;\;AMCL\left[F_{L:A\left(i\right)},C_{L:B\left(i\right)},OM_{A}\right]\;\;\;\to R_{G:A\left(i\right)},R_{G:B\left(i\right)}$8:  **end for**


## 3. Results

**Figure 8:**
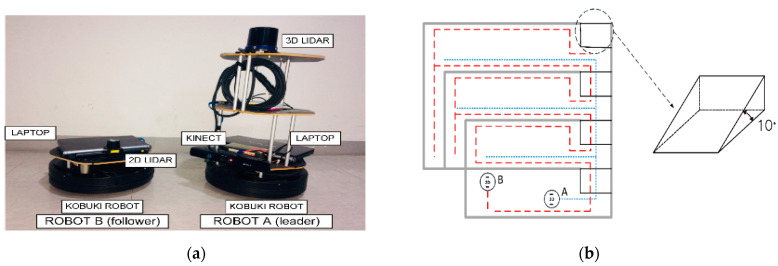

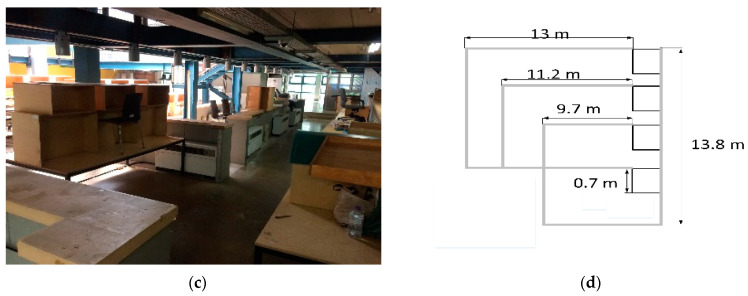
Experiment setting: (**a**) Two mobile robots and their parts, (**b**) the experiment location and corresponding robot paths, (**c**) Image taken from a corner of the experiment location, (**d**) experiment location diagram.

Our multi-robot system has two robots: Robot A (leader) and Robot B (follower). The leader robot is equipped with a 3D LIDAR scan, a Kinect camera, a Kobuki robot platform, and a laptop running Linux Ubuntu 14.04. The follower robot is equipped with a 2D LIDAR scan, a Kobuki platform, and a laptop running Linux Ubuntu 14.04. The master station uses Matlab with the robotics Tool Box 1.4 with an Intel i7 processor. Figure 8a depicts the two robots used for the experiment, and Figure 8b–d shows the experiment location. For the experiment, we use a university location provided with four ramps with 10-degree slopes. The ramps allow us to test the multi-robot performance in an uneven space. The leader robot collects the features using the process described in Section 2.2. The follower robot uses raw information from a 2D LIDAR scan.

The experiment coordinates systems are as follows: the world coordinate (x,y,z), leader robot $\left(x_{A},y_{A},z_{A}\right)$, and the follower robot $\left(x_{B},y_{B},z_{B}\right)$. The robot coordinates are always parallel to each robot’s velocity. Axes $z_{A}$ and $z_{B}$ are perpendicular to the soil. Both robots move with a linear velocity of 0.05 m/s. 

The 2D and 3D LIDAR sensor axes are concurrent with the follower and leader robots, respectively. The speed of both robots $V_{A}\;$ and $V_{B}$ are considered as non-slip speeds. The scanning frequency for the leader robot is every 0.5 m, and that of the follower robot is 0.1 m. The leader robot sampling period is Δt=1 s, and that of the follower robot is Δt=0.5 s. The follower robot has a shorter sampling period because 2D LIDAR scans are lighter than their 3D LIDAR counterparts. Both robots have enough time for data acquisition from LIDAR and odometry sensors employing the described sampling time. The proposed multi-robot system uses 3D point cloud information to identify features and recreate a 2.5D dense map. By contrast, the 2D LIDAR scans allow for fast localization on the even space. The ground truth for the robot localization was obtained using the odometer and the Inertia measurement unit (IMU) integrated into the robot. The information collected from these sensors was fused using the Extended Kalman Filter (EKF) within a ROS node.

**Figure 9:**
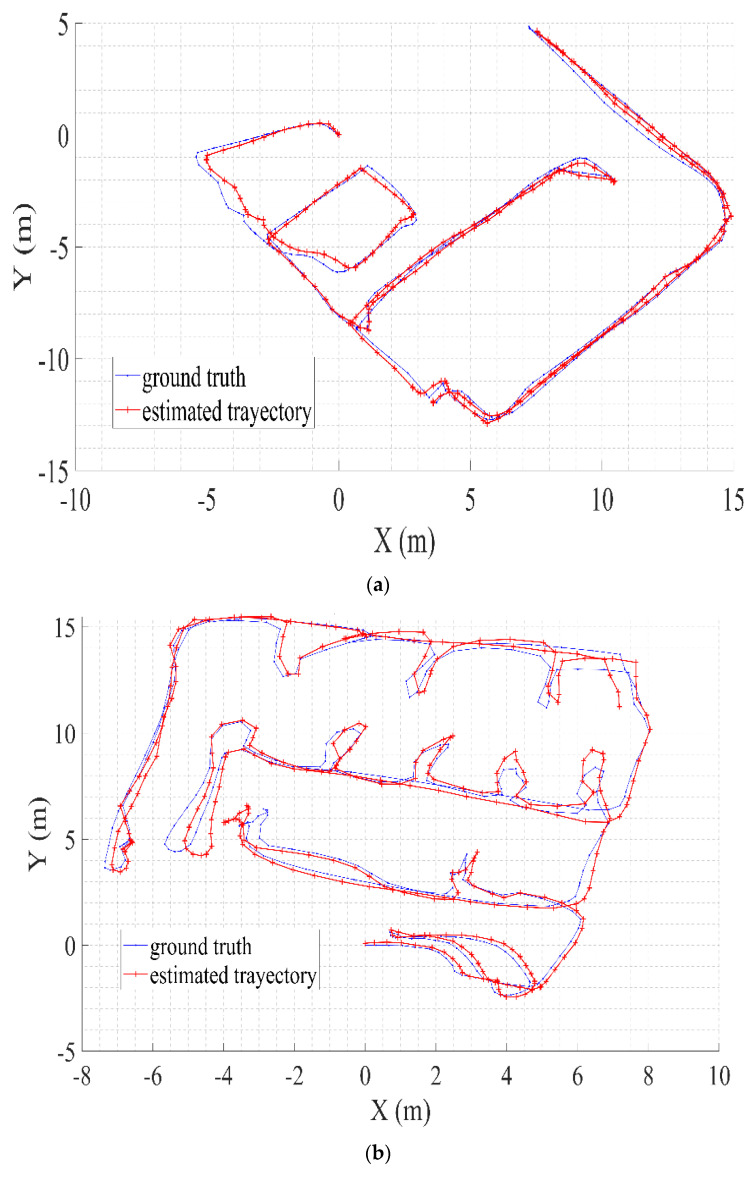

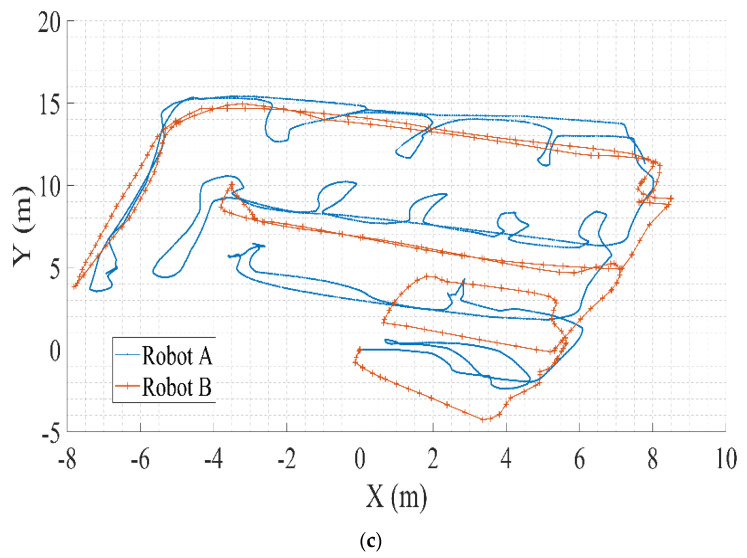
Multi-robot estimated trajectories and ground truth. (**a**) Leader robot (Robot A) trajectory. (**b**) Follower Robot (Robot B) trajectory. (**c**) Multi-robot trajectories.

We calculated the root-mean-square error (RMSE) in meters for the scan registration. Table 1 lists the RSME errors for both robots. Figure 9 shows the multi-robot estimated and ground-truth trajectories. Figure 9a,b are the trajectories of the leader and follower robots, respectively. The follower robot has a larger trajectory than that of the leader robot. As the leader robot uses a 3D LIDAR for exploration, his trajectory is shorter than that of the follower robot. Figure 9c shows the combined trajectory for both robots. Table 1 indicates that the leader and follower robots’ scanning errors are approximately 0.2 m in the X and Y axes, and this outcome acceptable for robot mapping and exploration.

The leader robot collects 3D point clouds for every pose and creates a 2.5D dense map. We used a box grid filter (BGF) with a voxel size of 0.1 m to down-sample each 3D point cloud. Once the robots completed the exploration, each robot generates a map. The leader robot generates a 2.5D dense map including all the collected 3D LIDAR point clouds. The 2.5D dense map created by the leader robot includes the floor points. The floor points are only removed for the generation of the occupancy grid map, as is described in Section 2.2. The follower robot recreates the 2D map using all the collected 2D LIDAR scans, the 2D LIDAR map was merged and filter using the process described in Section 2.3.

**Figure 10:**
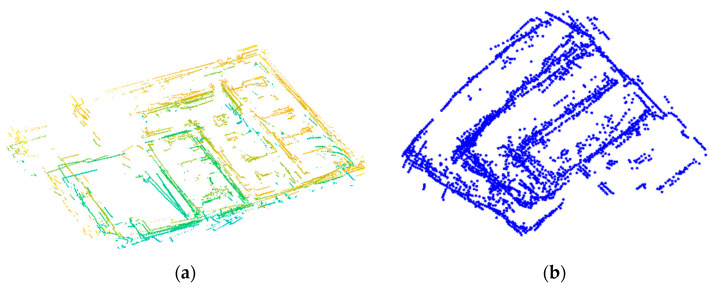
Maps generated by the multi-robot system. (**a**) 2.5D map generated by Robot A (Leader). (**b**) 2D map generated by Robot B (Follower).

Filtering the 3D point clouds enable quick registration, thereby maintaining accurate results. Figure 10a depicts the 2.5D map generated by the leader robot. Figure 10b shows the 2D map generated by the follower robot following the process described in Section 2.3. We compare our results with those of two state-of-art methods: generalized GICP and LOAM, to validate our method. As the proposed method was designed for multiple robots, we test the performance of each robot. To validate the trajectory obtained, we measure (in meters) the mean square error (RMSE) between the ground truth and the estimated trajectory. First, we employed two search algorithms: kd-tree and knearest-neighbor; which are the optimal ways to find the distance between two neighboring points. Second, once the closest neighbor pair was found, RMSE calculated the distance between two neighboring points. Table 2 and Table 3 show the localization error for each robot. LOAM and GICP are dependent on features extracted for even surfaces. As an uneven surface induces a sudden point cloud rotation, our method provides an accurate response for multi-robot localization on multi-level spaces.

This section may be divided by subheadings. It should provide a concise and precise description of the experimental results, their interpretation, as well as the experimental conclusions that can be drawn. Table 2 shows the error for the leader robot, for which our method error in axis Y is approximately 0.15 m that that of the GICP and LOAM. Table 3 shows the error for the follower robot. Our method errors in axes X and Y are approximately 0.2 m lower than those of the GICP and LOAM, and the processing time is also lower because we enhance the filtering and map merging for 2D scans. Table 4 shows the general errors in the multi-robot system, for which our method reduces the error in axes X and Y. The processing time of our technique is likewise lower than that of the GICP and LOAM. As our proposed method includes using a CNN, the 3D–2D exploration is versatile and allows for robot mapping within an optimal result. To provide a quantitative and qualitative comparison of the three methods, we only used MATLAB and ROS for programming. It was clarified in the results section.

## 4. Conclusions

This work allows the mapping and exploration of multi-level surfaces for multi-robots. Our mapping approach simplifies the global representation and localization using a merged occupancy grid map. The experiment results show a robust response in an environment integrated with multi-level surfaces. Our proposed 2D LIDAR scan merging method reduces the error localization around the x and y axes. The created Faster R-CNN has a robust response for detecting ramps in indoor environments. In future work, we shall extend larger indoor and outdoor mapping scenery.

## Figures and Tables

**Table 1:** Root mean square error for scan registration.

		Leader Robot	Follower Robot
Error (m)	X-Axis	0.26	0.22
	Y Axis	0.19	0.24
	Z Axis	0.31	0.86

**Table 2:** Leader robot localization errors for the proposed method, the generalized iterative closest point (GICP) technique, and the LIDAR and odometry mapping (LOAM) approach. The lowest values in the axis X, Y, Z, and consumed time are denoted in bold.

Leader Robot	Error (m)			Time (min)
	X	Y	Z	
Our Method	0.26	0.32	0.27	14.64
GICP	**0.18**	0.17	**0.17**	11.90
LOAM	0.25	**0.12**	0.19	**11.67**

**Table 3:** Follower robot localization errors for the proposed method, the generalized iterative closest point (GICP) technique, and the LIDAR and odometry mapping (LOAM) approach. The lowest values in the axis X, Y, and consumed time are denoted in bold.

Follower Robot	Error (m)		Time (min)
	X	Y	
Our Method	**0.02**	**0.05**	**12.10**
GICP	0.20	0.27	18.14
LOAM	0.20	0.27	18.14

**Table 4:** Total errors and times for a multi-robot system using our proposed method versus the general iterative closest Point (GICP) technique and the LIDAR odometry and Mapping (LOAM) approach. The lowest values in the axis X, Y, and consumed time are denoted in bold.

Multi-Robot	Error (m)		Time (min)
	X	Y	
Our Method	**0.28**	**0.37**	**26.74**
GICP	0.38	0.44	30.04
LOAM	0.45	0.39	29.81

## Data Availability

Not applicable.
